# Active Learning for the Discovery of Antiviral Polymers

**DOI:** 10.1002/marc.202500890

**Published:** 2026-02-06

**Authors:** Clodagh M Boland, Nhat Quynh Nguyen, Nathan RB Boase

**Affiliations:** ^1^ School of Chemistry and Physics Queensland University of Technology Brisbane Australia; ^2^ Centre for Materials Science Queensland University of Technology Brisbane Australia

**Keywords:** active learning, antiviral, machine learning, nanomedicine, polymer

## Abstract

The development of nanomedicines has long relied on scientific intuition within finite chemical space, but machine learning offers a data‐driven approach to explore far broader chemical landscapes. Advances in high‐throughput polymer synthesis and screening now enable the generation of datasets large enough to train predictive models to accurately link polymer structure with biological function. The next challenge is using machine learning to guide iterative development of nanomedicines through active learning. Here we demonstrate an active learning workflow for the design of antiviral polymers. Using molecular descriptors derived from polymer composition and monomer structure, a machine learning model was trained on an experimental dataset of antiviral polymer activity. The trained model was coupled with unsupervised clustering of monomers to explore chemical diversity and predict antiviral activity across a virtual library of up to 500,000 new polymers. By calculating the expected improvement function, active learning identifies optimal candidates for synthesis to efficiently explore chemical space and optimize antiviral activity. This communication highlights how machine learning and active learning can serve as practical, accessible tools for chemists and biologists to accelerate design of functional polymers and future nanomedicines.

## Graphical abstract

Machine learning and active learning are integrated to accelerate the discovery of antiviral polymers. Molecular descriptors derived from polymer composition enable predictive modeling of antiviral activity, while unsupervised clustering explores chemical diversity. The active learning workflow identifies optimal candidates for synthesis, demonstrating how data‐driven tools can guide polymer nanomedicine discovery.

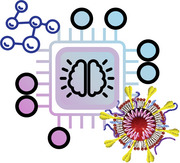

## 1 Introduction

The outbreak of COVID‐19 in March of 2020 demonstrated the widespread devastation that viruses can cause on a global scale. While the COVID‐19 pandemic demonstrated the fastest vaccine development in history, it was almost a year before the first doses were administered, with new, resistant strains emerging not long after [1]. The outbreak of COVID‐19 is just one example of how viruses can impact us. There are many lesser‐known viruses causing epidemics throughout the world for which vaccines have not been developed [2]. While vaccines are effective preventative strategies, there is still a need for therapeutics to treat infected patients to slow the spread or alleviate the symptoms associated with the infection. Some progress has been made in the development of combination drug therapies for treating viral symptoms however, like vaccines they are highly pathogen specific [3]. Broad spectrum antivirals offer a potential solution to the pathogen specificity problem, with polymers emerging as potential antiviral agents [4]. It has been shown that amphipathic polymers can be designed to interact with lipid membranes, like those of enveloped viruses, and in doing so reduce influenza and SARS‐CoV‐2 infection in human cells [5, 6]. These simple materials are promising candidates for broad spectrum antivirals that can be rapidly and efficiently deployed to treat a viral outbreak.

One of the major bottlenecks in early‐stage nanomedicine development is the rate at which novel structures can be synthesized and tested. In the past, polymer design has taken a brute force approach, requiring time consuming and costly experimentation during which researchers don't even come close to exploring the entire chemical space available to polymers [7]. However, due to the advancement in oxygen tolerant polymerization methods, we are now seeing the emergence of high throughput synthesis and screening techniques which allow us to rapidly explore polymer design space [8, 9]. To support these methods, we need data science tools to help guide polymer design to target valuable areas of chemical space to achieve the goal at hand [10].

The use of high‐throughput synthesis in polymer science enables the generation of large data sets, which in turn facilitates the use of machine learning (ML) to interpret these large datasets [11]. ML can be used to develop mathematical models that can correlate specific structural features of polymers to their physical, chemical, or biological properties [5, 12, 13]. The symbiotic relationship between high‐throughput synthesis and ML allows for the development of highly automated polymer discovery workflows and self‐driving laboratories [14]. A simple ML workflow involves data collection, model training on a training dataset, and model evaluation on a test dataset. ML has already been used to model polymer suitability for biomedical applications, for example, gene delivery, magnetic resonance imaging (MRI) contrast agents, and antimicrobials [15, 16, 17, 18, 19]. As an example, a decision tree model was trained on a dataset consisting of 157 acrylate copolymers and their antimicrobial properties [17]. Through the use of SHapley Additive exPlanations (SHAP), they determined that cationic and hydrophobic characteristics of polymers have a significant effect on the polymer's antimicrobial properties, matching similar findings for antimicrobial peptides [19]. Reversible addition‐fragmentation chain transfer (RAFT) polymerization has been combined with high‐throughput characterization and a random forest model to predict gene transfection efficiency of a library of 43 copolymers, concluding based off SHAP analysis that polymers composed of cationic and a moderate amount of hydrophilic monomers exhibit the greatest potential for transfection [15]. These studies demonstrate that ML can minimize time, cost, and labor in polymer nanomedicine development [15].

Due to the vast chemical space occupied by polymers, one of the major challenges when using machine learning in polymer science is ensuring that our model can generalize to unseen structures [20]. Active learning is one approach that can be used to dynamically optimize our model. This works by having our model make predictions on unseen data and then calculating the uncertainty of those predictions. The result leaves us with a matrix of values for the estimated uncertainty and the predicted value itself. Selectively choosing values from this matrix to experimentally validate can help us to improve the model while minimizing time and cost [21]. An excellent example of the efficiency of active learning was demonstrated by predicting ^19^F MRI contrast agents while only experimentally synthesizing <0.9% of the compositional space [16].

While ML has already proven to be a very useful tool in polymer science, there is still room for improvement to capitalize on all the benefits ML has to offer. One of the fundamental factors underpinning model performance is the quality and quantity of our dataset [7, 22]. To date, generating a large dataset has relied heavily on the use of literature and polymer databases, all of which can compromise the quality of data due to lack of consistency across studies [23]. The optimal way to generate a robust dataset is through experimentation, and high throughput synthesis gives us the opportunity to do that. Although it is unfeasible to synthesize hundreds of thousands of polymers, the combination of high throughput synthesis and active learning allows us to strategically build a diverse but concise dataset that well represents the broad chemical space occupied by polymers while minimizing experimentation and keeping our dataset to a computationally reasonable size [24].

Previously, our team used 10 methacrylates to synthesize a library of 263 polymers and screened them for antiviral activity, training a random forest model to link copolymer composition with antiviral performance [5]. In this work, we build upon that foundation to develop an active learning workflow in which machine learning models are used to predict the antiviral activity of new polymers constructed from previously untested monomers. We hypothesize that active learning can efficiently explore diverse chemical space and identify polymers with enhanced antiviral activity. To achieve active learning on diverse polymer compositions, molecular descriptors were used to represent the features of the polymers, and train multiple ML regression algorithms. After selecting the best performing model, we employed a random combinatorial approach to generate ∼500,000 new copolymer structures from a library of 119 commercially available methacrylates. Active learning was used to identify polymers for future development with either a focus on enhancing the model by exploring new chemical space or exploiting the model for optimal predicted outcomes. This work demonstrates an active learning workflow that can guide iterative design‐train‐test cycles for the discovery of polymer antivirals, and more broadly illustrates the applicability of these data‐driven methods to the rational design of functional materials and nanomedicines

## 2 Results and Discussion

### 2.1 Feature Generation for Antiviral Copolymers

In our previous work, we discovered new broad spectrum antiviral polymers using a high‐throughput approach to screen copolymers of known antimicrobial methacrylates, in combination with biocompatible and hydrophobic methacrylates. This enabled us to develop a machine learning model to link polymer composition with antiviral activity [5]. Based on the number of methacrylates used (10) and known limits to monomer composition for solubility and toxicity, we had screened an estimated 10% of the available chemical space and discovered most of the efficacious combinations of these monomers. To enable discovery of new antiviral polymers would require a model that could be generalized across new monomers. To achieve this required training a machine learning model on computable molecular descriptors of the copolymers as features, rather than defined polymer compositions of a set library of monomers.

To calculate the molecular descriptors for the monomers and polymers in this work, a combination of open source tools were used including Python (language), Jupyter Notebook (interactive environment), RDKit (chemoinformatics toolkit) and Mordred (chemical descriptor calculator). It was intended to develop a semi‐automated approach, to simplify generating descriptors for large libraries of unknown monomers (for use in future active learning). The full code has been made publicly available [25], and a summary of the workflow provided (Figure S1). As an overview of the key steps involved: (1) manually creating a list of monomers with their Chemical Abstracts Service (CAS) numbers, (2) automatically generating canonical simplified molecular input line entry system (SMILES) strings by searching PubChem using the CAS number (missing SMILES strings could be manually added) [26], (3) ‘hydrogenation’ of the vinylic functional group, and (4) generation of the mol file and calculation of the chemical descriptors using Mordred. ‘Hydrogenation’ of the vinylic unit was performed by modifying the SMILES string to replace the alkene (C = C) with an alkane (C‐C), to better emulate the properties of the monomers when polymerized. Mordred was selected as the molecular descriptor calculator for this work as it is open source, provides a large library of potential descriptors (1825), and can perform robust and fast calculations [27]. In this work, 3D descriptors were not included because they are computationally expensive and in the case of long chain polymers, the results are highly dependent on the polymers’ environment [28]. This provided a pool of 1613 potential descriptors. After data cleanup to ensure descriptors were calculated correctly for each sample and had unique values, there were 786 descriptors for the methacrylates used in the antiviral polymers, to train an ML model.

### 2.2 Data Preparation and Training of Machine Learning Models

**FIGURE 1:**
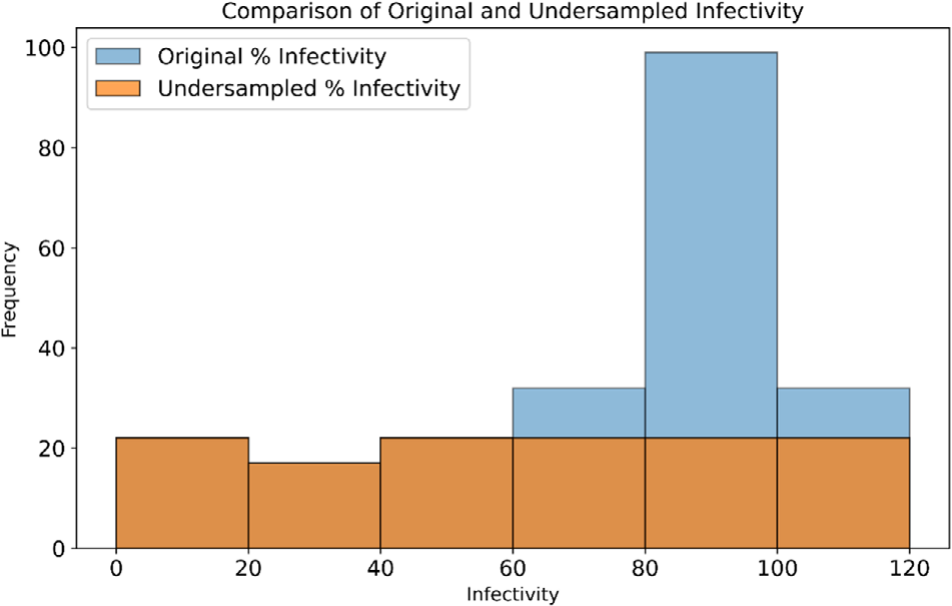
Normalization of training dataset using an under‐sampling approach to improve the trained model's accuracy at predicting antiviral copolymers (infectivity < 20%).

From our previous work we had access to a dataset of 263 different copolymers made up of 10 methacrylates that had been assessed for antiviral activity, using an in vitro immunoplaque assay [5]. Samples with low values for infectivity (nominally <20%) are considered antiviral in screening. This dataset was used to train a machine learning model on molecular descriptors of the copolymers, rather than polymer composition. This would allow for prediction of new untested monomers. The existing dataset was heavily skewed toward polymers that are unlikely to be antiviral (infectivity >20%), particularly with almost no effect (>80% infectivity). When regression models were trained on the full dataset, they led to poor generalization with the test data, particularly in the low infectivity range, which was of most importance to this project (Figure S1). To improve the model's accuracy to predict copolymers with low infectivity, the dataset was normalized using a straightforward under‐sampling approach, to provide a similar number of samples across bins and the distribution of results (Figure 1). Samples were only removed from bins >40% infectivity, to preserve the maximum amount of data for polymers with the desirable property of low infectivity. After removing duplicate copolymers with identical compositions and then randomly removing samples until the bin sizes were approximately equal to the size of the 0–20% bin, the dataset had 127 unique samples remaining.

The next stage of data preparation required calculation of descriptors for the random copolymers from the monomer descriptors. This was achieved by using a linear combination of the chemical descriptor for each monomer (m) weighted by the molar fraction of each monomer (χ) given by: [29]

(1) $$m_{copolymer}=m_{1}\;\chi_{1}+m_{2}\chi_{2}\cdot \cdot \cdot +m_{n}\chi_{n}$$

In practicality this was achieved by the dot product of a matrix of copolymer composition values in mol% against a matrix of the previously computed monomer descriptors [30]. After clean‐up, the dataset had 678 valid descriptors for the 127 samples. This dataset was then split into test (20%, 25 samples) and train (80%, 102 samples) datasets prior to any feature reduction to prevent data leakage to the model. A group‐shuffle‐split approach was used to ensure samples of identical composition were contained in either test or train fractions, again to prevent data leakage.

**FIGURE 2:**
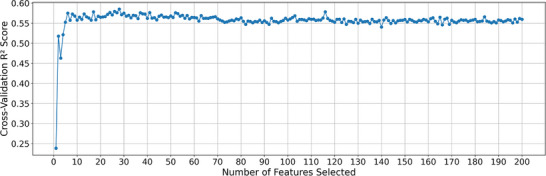
Recursive feature elimination with cross‐validation (RFECV) was used to identify the optimal subset of molecular descriptors for model training and to reduce feature dimensionality.

To simplify the machine learning model, and increase efficiency of training, it is necessary to reduce the number of trained features. In this work this was achieved by first performing univariate feature reduction to remove features that showed no correlation with infectivity. This provided a rapid method to reduce the number of features, with the top 200 correlating features arbitrarily selected. More accurate recursive feature elimination with cross fold validation (RFECV) was performed using a random forest model on the remaining 200 features (reduces computation time <10 min) to understand how each feature contributes to the accuracy of the model (Figure 2) [31]. This analysis showed it was possible to select just 17 features to explain most of the variability in our model greatly reducing the complexity of our dataset (Table S1).

**FIGURE 3:**
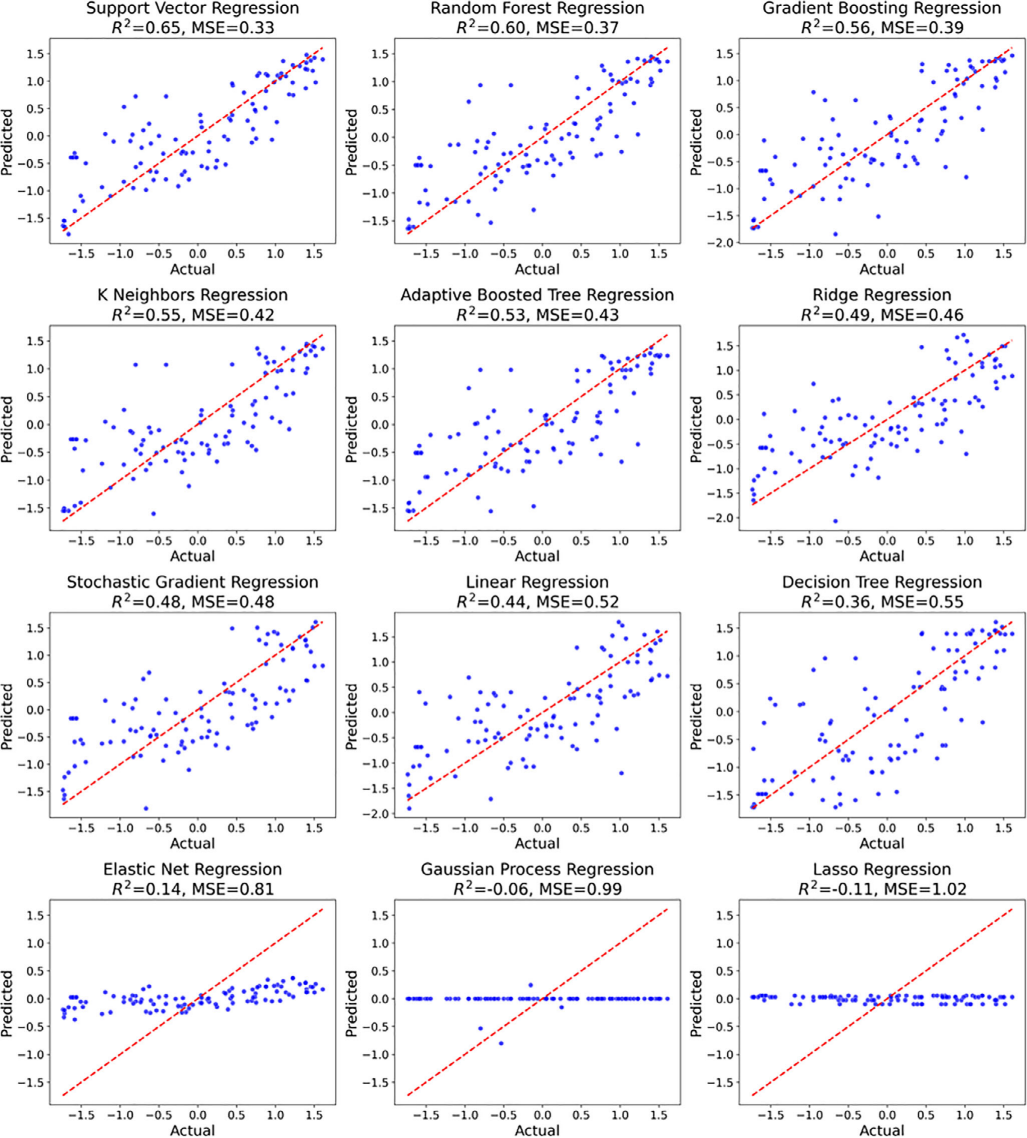
Training and evaluation of 12 regression models from Scikit‐Learn using polymer molecular descriptors to predict viral infectivity, illustrating model performance across different algorithms.

With a workable dataset of molecular descriptors for the synthesized antiviral copolymers, it was now possible to train a machine learning model. Scikit‐Learn was used to first scale the data using the standard scaler, then a range of out of the box regression models were tested using default hyperparameters (Table S2). From this analysis it was clear that support vector regression (SVR) and random forest regression (RFR) already performed quite well (R^2^> 0.6), with gradient boosting regression (GBR) as third highest performing model (R^2^ = 0.56) (Figure 3; Figure S2 and Table S1). This represents a mixture of kernel‐based (SVR) and different decision tree approaches (RFR, GBR). Hyperparameter tuning for these models was investigated to see if model performance could be improved and decide on a final optimal model.

**FIGURE 4:**
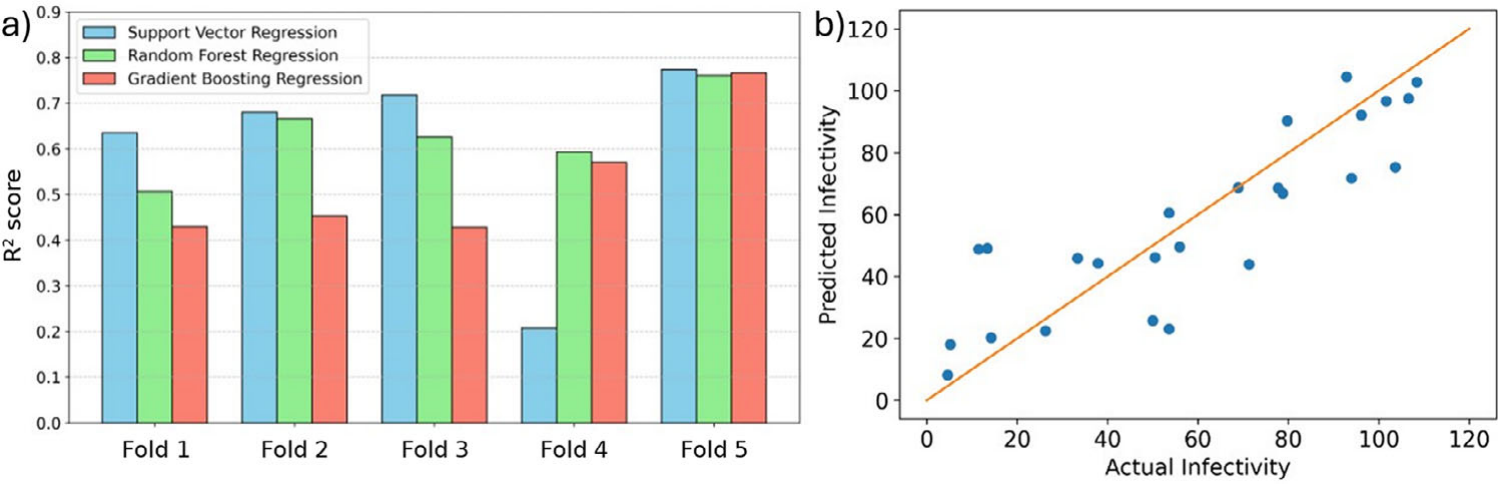
(a) Generalizability of the top three models evaluated using a nested five‐fold cross‐validation on the training dataset after hyperparameter tuning. (b) Performance of the final random forest regression model on the test dataset, showing accurate predictions (R^2^ = 0.74, MAE = 13%, RMSE = 17%).

Each machine learning model has specific hyperparameters that can be tuned to improve the performance of the model. Hyperparameter tuning was conducted on the top three performing models: support vector regression, random forest regression, and gradient boosting regression (Table S4). To do this, a nested five‐fold cross‐validation approach was used to improve robustness and generalizability of the final models [32]. Again, a group shuffle split approach was used for setting up the five folds, to prevent data leakage across the folds. Given the relatively small number of samples in each fold (∼20), concentration of duplicate samples into a single fold did affect generalizability of some models tested in this work. The analysis showed clearly that while on average across the training data all models performed well, RFR was the best performing model across all folds (R^2^> 0.5, Figure 4a), suggesting low chance of overfitting and good generalizability of the model. SVR performed high across 4/5 folds (R^2^> 0.6) but performed very poorly in fold 4 (R^2^ ∼0.2), while GBR had lower correlation across all five folds compared to RFR. Based on this analysis, RFR was selected as our optimal model for our dataset. Training of the model against the full training dataset led to a high performing model, with reasonably high correlation (R^2^ = 0.74) and low error in predicted infectivity (mean absolute error (MAE) = 13%, root mean square error (RMSE) = 17%) (Figure 4b). Importantly, these levels of error and uncertainty are in line with comparable studies, suggesting that the observed fit is appropriate given the inherent noise and variability in infectivity measurements [33]. The accuracy of the model can potentially be further improved using an iterative active learning approach, introduced later in this article [34]. When we specifically evaluated the outlier polymers for which the model's predictions had the largest absolute error, we found that all the outliers contained charged monomers (Table S7). This suggests our current polymer representation and model training is not accurately capturing this specific subset of monomers, and requires further refinement in future iterations.

**FIGURE 5:**
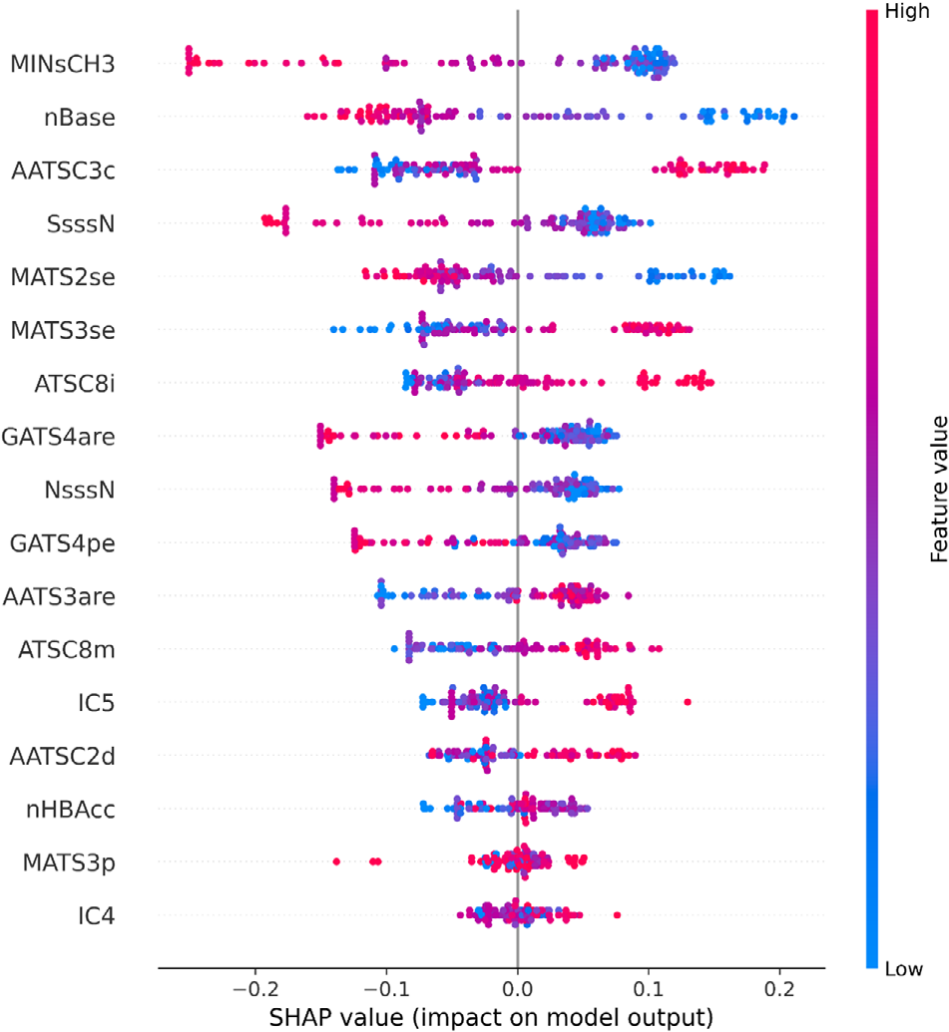
Contributions of molecular descriptors to model predictions, quantified by SHAP analysis, highlighting the most influential features. (Negative SHAP value is lower infectivity and indicates more antiviral effect).

To understand how the model uses the features to predict infectivity, SHAP values were computed for each of the selected features in the model. From the SHAP analysis, the values for features that contribute highly to the model (top of Figure 5) correlate strongly with the impact it has on the model, demonstrating reliability of the model. The features are the calculated molecular descriptors, and some are easily interpretable (eg. nBase = number of basic amino groups, nHBAcc = number of hydrogen bond acceptors), and others are more esoteric chemometric parameters (Table S1 provides an overview of all descriptors selected). These span several types including atom‐type counts and E‐State descriptors (e.g. NsssN, SsssN, MINsCH3) capturing the presence and electronic environment of key functional groups, autocorrelation descriptors (AATS, ATSC, AATSC) and spatial autocorrelation descriptors (MATS, GATS) describing how atomic properties such as electronegativity, charge, mass, and ionization potential are distributed along the polymer backbone, and information content descriptors (IC4, IC5) representing local topological complexity. As was seen in previous research, it is a complex interplay of topology, charge characteristics and polarity that drive antiviral polymer function [5]. Although interpretability provides insight into structure–activity relationships, active learning allows for the identification of new promising antiviral candidates without requiring the user to fully understand the underlying descriptors. This analysis also highlights the value of feature elimination, with the last two features of even this small sub‐set having poor correlation between feature value and model performance. These low correlating features have little impact on model output and could be eliminated from future model training.

### 2.3 Using Machine Learning to Predict New Antiviral Polymers

A random forest regression model was successfully trained to predict antiviral activity from molecular descriptors of copolymers. The next step was to determine whether this model could generalize to unseen polymers and be applied in an adaptive learning workflow for new candidate discovery. The challenge with using regression models is that while they are good for accurate predictions and are computationally economical, they are limited in their ability to generate new theoretical structures from the model [32]. To overcome this challenge, a random combinatorial algorithm was trialed to create new hypothetical copolymers, compute their molecular descriptors, and then test these theoretical structures against the trained model. To demonstrate this workflow a list of 119 commercially available methacrylate monomers was selected (Table S5), keeping the polymer backbone the same as our pre‐trained model for simplicity in this first iteration. These monomers represented diversity in their side chains, from hydrophilic to hydrophobic, charged and uncharged, cyclic and aromatic, to fluorinated and siloxanes. Using a random generation approach on linear combinations of all 119 monomers and a maximum of four comonomers per copolymer would require large computational time and produce an inordinate number of functionally useless materials. Prior work had identified that if polymers possessed combinations of a hydrophilic monomer, hydrophobic monomer and basic amino group, they had a high likelihood of being antiviral [5]. So classifying monomers into similar classes, and selecting one monomer per class to build the copolymer was one approach to reduce the chemical space to be interrogated. While this could be done by manually classifying each monomer based on chemical intuition of the researcher, it was decided to take an unsupervised approach, to enhance efficiency over large monomer sets and reduce researcher bias into the active learning workflow. Dimensionality reduction of the high‐dimensional data was attempted using Uniform Manifold Approximation and Projection (UMAP) and unsupervised clustering of similar monomers by k‐means clustering.

**FIGURE 6:**
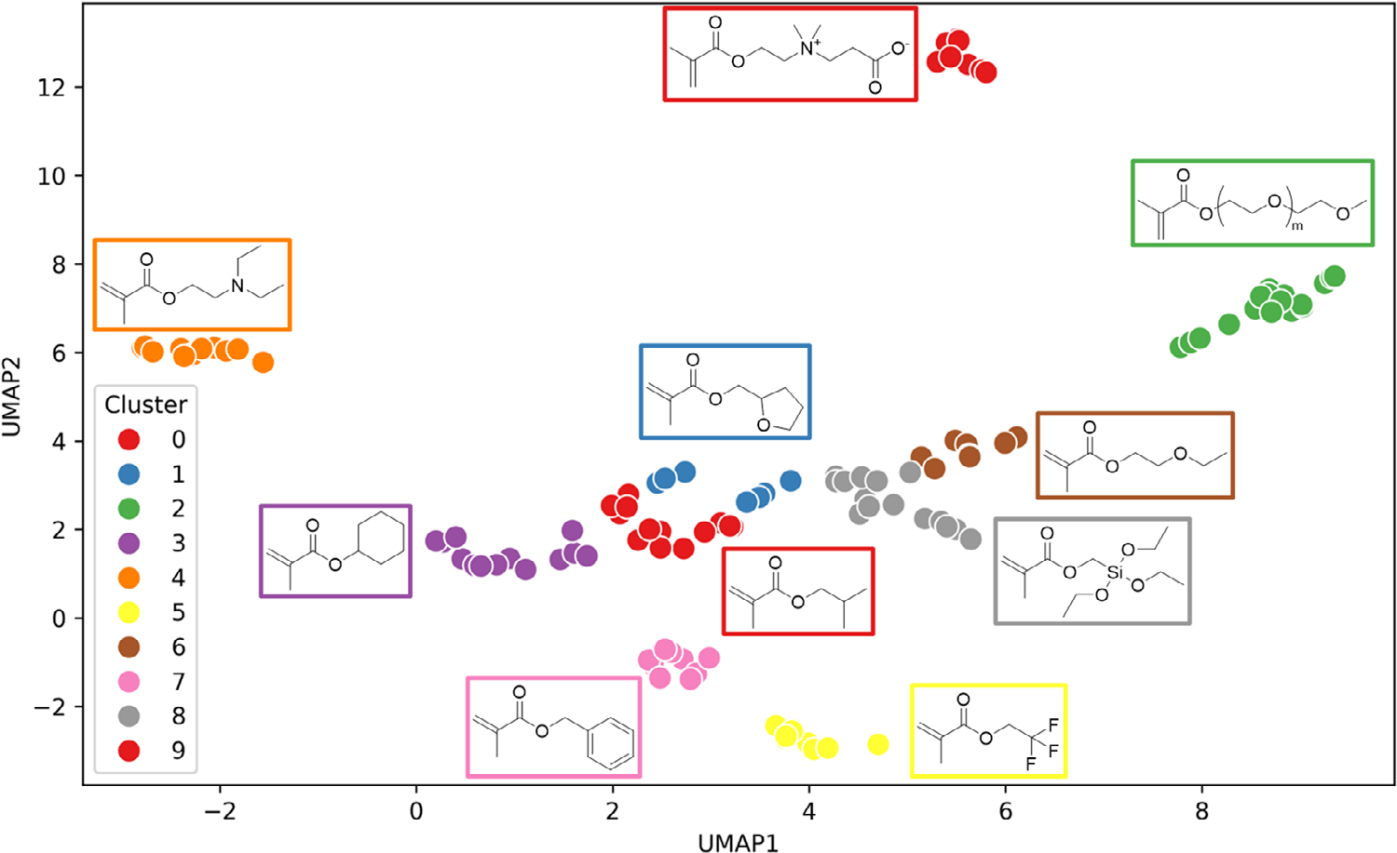
Unsupervised learning was used to cluster methacrylates into structurally similar groups using a combination of UMAP dimensionality reduction and k‐means clustering. One exemplary structure is shown for each cluster.

To cluster similar monomers together, molecular fingerprints and molecular descriptors were computed using RDKit and Mordred. Morgan fingerprints were computed for each monomer. Through some optimization, it was found that using a radius of 2, length of the binary fingerprint vector as 1024, and atom identities provided the best clustering. Clustering of the monomers was first attempted using only molecular fingerprints, but this led to inefficient clustering and clustering of monomers that intuitively should be clustered separately (long hydrophobic alkyl chains often clustered with short tertiary amines). Through experimentation, a small subset of interpretable molecular descriptors relevant to the monomer library were found to be beneficial to assisting monomer clustering, including SLogP, TopoPSA, nN, nO, nRot, nHBDon, nHBAcc (Table S6). A combined feature matrix was computed using molecular fingerprints, and the molecular descriptors with a two‐fold weighting to balance the influence each component had on the model. This combined feature matrix was used to optimize the hyperparameters for UMAP reduction (Figure S3), with 5 neighbors and a minimum distance of 0.1 ultimately selected. Finally for k‐means clustering, the optimal number of clusters was determined by computing within‐cluster sum of squares (WCSS), silhouette score and Davies‐Bouldin Index for k‐means clustering. Comparing these different scoring methods, 10 clusters were selected, leading to efficient and robust clustering of the methacrylate monomers in our library (Figure 6; Table S6). Molplotly was useful to create an interactive interface for model evaluation, actively showing the monomer structure in each cluster when scrolling across the plot (Figure S5).

With a library of potential monomers clustered into similar groups, it was now relatively simple to generate new random copolymer structures. A random combinatorial algorithm was written that would create new polymers, selecting 1–4 monomers and varying comonomer composition in 10 mol% increments. A limit to the maximum total number of structures was provided to limit computational time, and a smaller limit to the maximum number for any given monomer was set, to maximize diversity of monomers sampled. It was found to be more computationally economical to generate all combinations randomly, then remove duplicates, than to try and create unique copolymers at every given step. This led to a library of 500,000 copolymers generated in less than a minute (∼70,000 duplicates removed afterwards). As with the original trained model, molecular descriptors were easily calculated for all theoretical structures by computing the dot product of the copolymer composition matrix and the monomer descriptor library (1.2 h computation time on standard PC). While it was simple to generate many unique polymers, many of these may be functionally useless, even if they are predicted to be antiviral. For example, if the polymers are not water soluble, they cannot be used in biological testing. To reduce the chemical space the SLogP descriptor was computed for all polymers synthesized in the original antiviral dataset (min = −132, max = 192, mean = 66 ± 93), as all these molecules were known to be water soluble at a concentration suitable for antiviral testing. A limit of the maximum SlogP + 10 of the synthesized library was then applied to the theoretical library, to minimize the likelihood of predicting insoluble materials, leaving ∼230,000 candidates. This combinatorial approach demonstrates a straight‐forward and easy to implement approach for novice users, without the need for using more complex artificial intelligence (AI) approaches.

**FIGURE 7:**
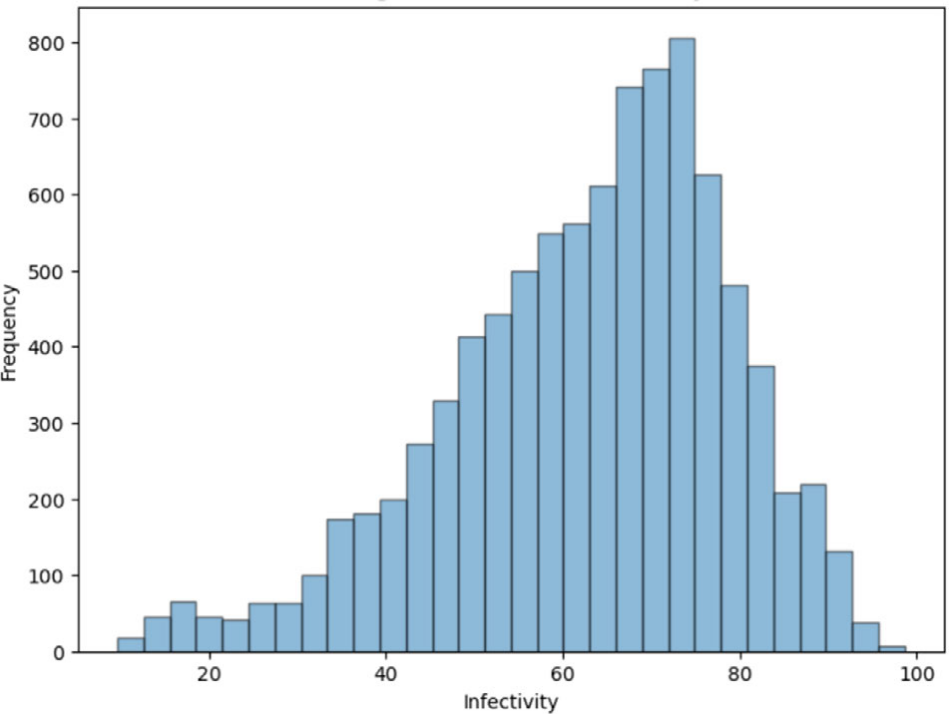
Distribution of predicted viral infectivity values for a combinatorial library of ∼230,000 polymers, as estimated by the trained random forest regression model.

The features of the final library of selected theoretical polymers were scaled, and their antiviral activity predicted using the trained RFR model (Figure 7). From high‐throughput testing of antiviral polymers, a nominal threshold of less than 20% infectivity was found to be a reliable predictor of antiviral activity of synthesized polymers [5]. Considering the trained model had an uncertainty of approximately 15% (MAE = 13%, RMSE = 17%), a threshold of 35% could be considered for identifying likely antiviral polymer candidates. Therefore, while the vast majority of randomly created structures predicted were not likely be antiviral, many promising antiviral candidates were predicted. This was a successful outcome given the random combinational approach to polymer generation used, and the straightforward implementation.

### 2.4 Active Learning to Discover New Antiviral Polymers

The final step in the workflow was to decide what polymers to make next from this large library of candidates. Active learning is a dynamic method that can be used to both enhance the applicability domain of the model (exploration) or optimizing the output of the model (exploitation). As in this work, where a new model is being developed on a new dataset, it is often desirable to balance exploration and exploitation to optimize both outcomes over cycles of experimentation. Computing the expected improvement (EI) acquisition function can allow for a single algorithmic framework to be used, that can tune the exploration‐exploitation tradeoff: [14]

(2) $$\mathrm{EI}\;\left(x\right)=\left(y_{\mathrm{best}}-\hat{y}\left(x\right)-\xi \right)\;\Phi \left(z\right)+\sigma \left(x\right)\;\phi \left(z\right)$$

where for a minimization task (smaller infectivity is desirable):

(3) $$z=\frac{y_{\mathrm{best}}-\hat{y}\left(x\right)-\xi }{\sigma \left(x\right)}$$

EI(*x*) = Expected Improvement at point x

$y_{\mathrm{best}}$= current best observed value

$\hat{y}\left(x\right)\;$= predicted mean at x

σ(x)= predicted standard deviation at x

ξ= exploration parameter (controls trade‐off between exploration and exploitation)

Φ(z)= standard normal cumulative distribution function

ϕ(z)= standard normal probability density function

Early cycles of experimentation can favor synthesizing polymers with higher uncertainty in a model (ξ > 0.3), allowing for retraining the model to become more generalizable across a wider applicability domain. Later stages of development can then switch focus to exploitation (ξ = 0), finding optimal solutions that are now robust and representative of diverse chemical space. This iterative process of retraining the model with carefully selected datapoints should lead to an improvement in Random Forest Regression model's error metrics.

**FIGURE 8:**
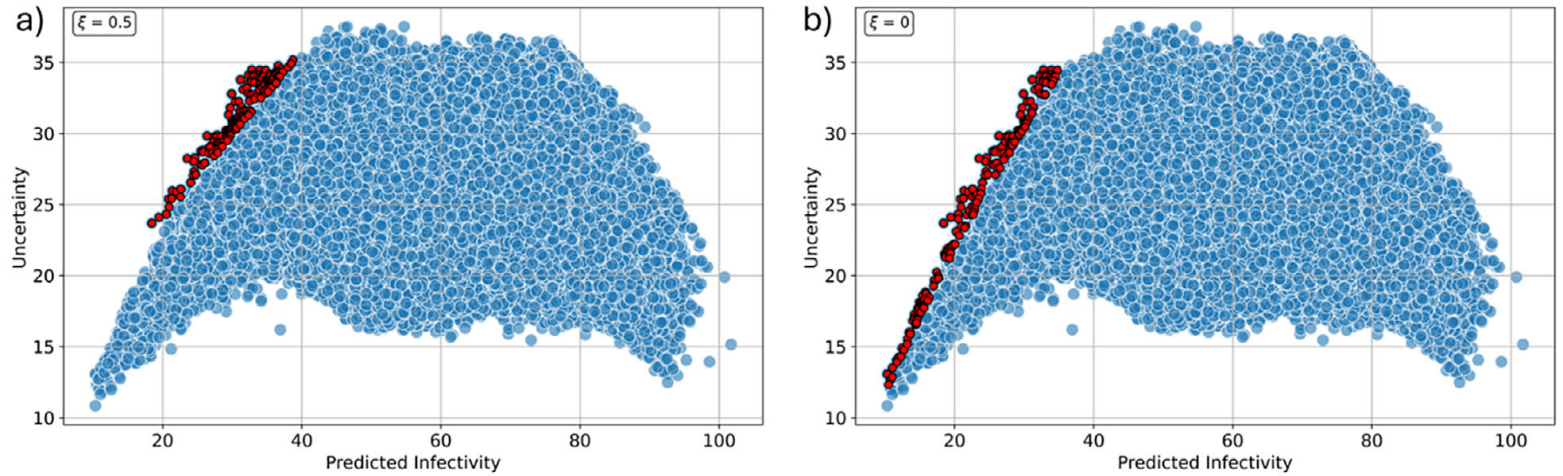
Predicted viral infectivity versus random forest regression uncertainty for the predicted polymer library. The top 200 candidates using the expected improvement (EI) function with (a) ξ=0.5 or (b) ξ=0 highlighted in red, illustrating how active learning balances exploration (a, high uncertainty) and exploitation (b, low predicted infectivity).

To compute EI, models that provide measurements of uncertainty are needed. Gaussian process regressors are commonly used for this task, as uncertainty is automatically calculated in these models, but these models were not applicable to this dataset (Figure 3) [35]. Prediction uncertainty of the random forest regression model was estimated from the variance of predictions across the individual decision trees in the random forest ensemble (bootstrap‐derived variance) [36]. Using this uncertainty estimation, it was possible to calculate exemplary EI values for each polymer candidate, with either a focus on exploration (ξ = 0.5) or exploitation (ξ = 0). The top 200 EI values for each case were then identified out of the whole dataset, visualizing the difference between an exploration strategy (Figure 8a, favoring model uncertainty), or exploitation to optimize the output (Figure 8b, favoring lowest infectivity). This provides two complementary strategies that can be used in the iterative discovery of new polymer antivirals.

The next step in this project is applying the active learning workflow in iterative design‐test‐train cycles to fully evaluate its utility for developing potent antiviral polymers. Given the vast number of potential structures and the need for a diverse and substantial dataset, the integration of automation or self‐driving labs into the research program will be necessary [14, 24]. Currently the model is limited to methacrylate monomers, but future work aims to integrate broader classes of vinylic monomers and eventually other polymer classes, potentially through unsupervised learning of side chains or new strategies to account for variations in backbone architecture. Another solution to be explored is using generative artificial intelligence (AI), rather than combinatorial libraries of molecular descriptors [37]. Generative AI may be able to more accurately capture the structural elements of the polymer that drive antiviral function, and enable more efficient prediction of highly efficacious and functional polymer antivirals. A major challenge for employing generative AI is the need to encode the polymer structure in a machine‐readable format and requiring use of more complex machine learning methods, limiting its practicality for non‐specialist researchers. Future work aims to understand the mechanism of action of these antiviral polymers. Previous work by our group has demonstrated that the lead candidate's antiviral activity involves an interaction with the viral lipid membrane. We are currently investigating the nature of this interaction, and it is hypothesized that there is selective action on the viral vs cell membrane meaning these polymers have high antiviral activity while having low cytotoxicity [5].

## 3 Conclusions

The integration of machine learning and active learning offers a practical approach to accelerate the discovery of antiviral polymers, and other nanomedicines more broadly. This study demonstrates an integrated workflow that trains machine learning models on molecular descriptors of copolymers to effectively predict antiviral activity. By calculating molecular descriptors for individual monomers and applying unsupervised learning methods like UMAP and k‐means clustering to group them, the workflow enables development of generalizable models that can be applied across diverse copolymer compositions. Random forest regression was applied to the existing dataset to train an accurate model capable of predicting antiviral activity of both known polymers and new polymers generated through a random combinatorial algorithm. Integrating active learning into the workflow facilitates future iterative rounds of development that will not only lead to the discovery of new antiviral polymers but optimize their composition for maximum activity. Beyond antivirals, this workflow is intended to be easy to understand and use, offering translation to other polymer nanomedicines. Our work highlights that data science tools can be readily adopted by chemists, biologists and materials scientists to accelerate the discovery of new nanomedicines.

## 4 Methods

### 4.1 Computational Details

All computation was carried out using Python programming in the Jupyter notebook environment, using open source packages including: Pandas [38], Numpy [39], Scikit‐Learn [40], RDKit [41], Matplotlib [42], and Shapley Additive exPlanations (SHAP) [43]. The full code and package versions used in this work are available through QUT Research Datafinder [25]. All model development, training and analysis was performed on a standard personal laptop with an Intel I5‐1135G7 processor (quad core, 2.40 GHz), 8.0 gb DDR‐4 RAM, and Intel IRIS Xe integrated graphics card.

### 4.2 Data Description

Experimental data for antiviral activity of polymers was acquired previously [5], and is available through QUT Research Datafinder [44]. The available dataset reports polymer composition by weight percent (wt.%) of each monomer, and the % infectivity (defined as plaque count for each polymer divded the plaque count in the virus control multiplied by 100). A low % infectivity is more effective reduction in viral infection of cells, with a nominal 20% infectivity used as a benchmark to label a polymer antiviral during high‐throughput screening. For model development, polymer compositions were converted from wt.% to mol% of each monomer to generate molecular descriptors used in machine learning analyses. Datasets and features were scaled prior to model training using the StandardScaler function of Scikit‐Learn.

## Author Contributions

The manuscript was prepared through contributions of all authors, with specific contributions recognized below using the CRediT framework. C.M.B. performed in writing – original draft, writing – review and editing. N.Q.N. performed in formal analysis, software. N.R.B.B. performed in conceptualization, data curation, formal analysis, funding acquisition, project administration, software, supervision, visualization, writing – original draft, writing – review and editing.

## Funding

Data collection in this work was supported by the Assistant Secretary of Defense for Health Affairs endorsed by the Department of Defense, in the amount of $213,313 through the Peer Reviewed Medical Research Program under Award Number W81XWH‐22‐1‐0106. Opinions, interpretations, conclusions, and recommendations are those of the author(s) and are not necessarily endorsed by The Assistant Secretary of Defense for Health Affairs endorsed by the Department of Defense.

## Conflicts of Interest

The authors declare no conflicts of interest.

## Supporting information




**Supporting File**: marc70227‐sup‐0001‐SuppMat.docx.

## Data Availability

The data that support the findings of this study are openly available in [QUT Research Data Finder] at [https://doi.org/10.25912/RDF_1762215850882], reference number [43353].
