# Supplementary material for: Active Learning for the Discovery of Antiviral Polymers
Source: Macromol Rapid Commun. 2026 Feb 6;47(8):e00890. doi: 10.1002/marc.202500890 (PMC13087829; doi:10.1002/marc.202500890)
Supplement: Supplementary file 1 — Supporting File: marc70227‐sup‐0001‐SuppMat.docx. [file MARC-47-e00890-s001.docx]

Supporting Information

Active Learning for the Discovery of Antiviral Polymers

Clodagh M Boland, Nhat Quynh Nguyen, Nathan RB Boase*


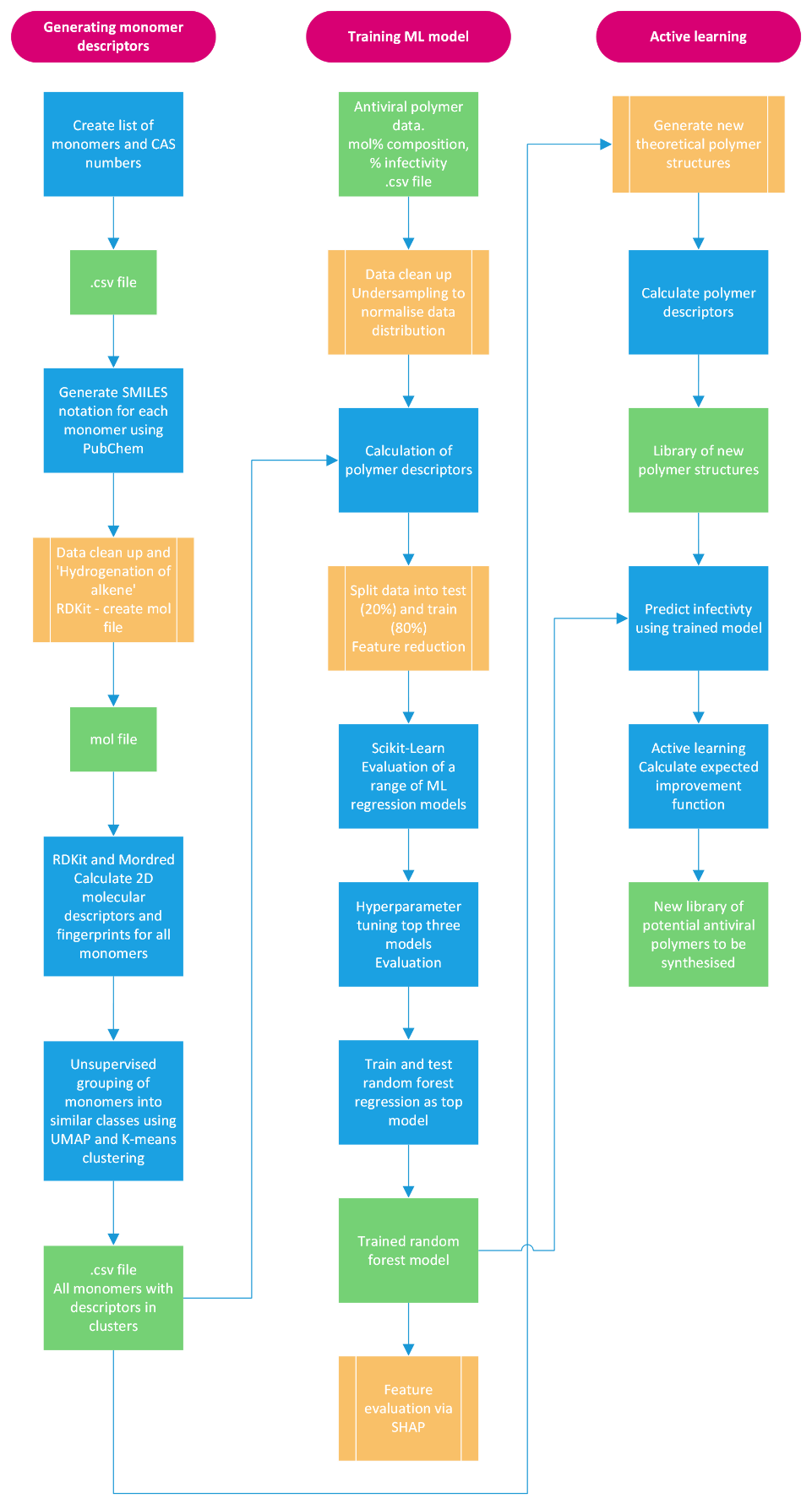


Figure S1. Flow diagram demonstrating the three key workflows used in this work including (1) generating monomer descriptors and unsupervised clustering, (2) training a machine learning model on existing antiviral polymer data, and (3) using active learning to predict new polymers that could potentially be antiviral. Blue boxes indicate key steps in each workflow, orange boxes indicate data processing or manipulation, and green boxes indicate outputs of the workflow.


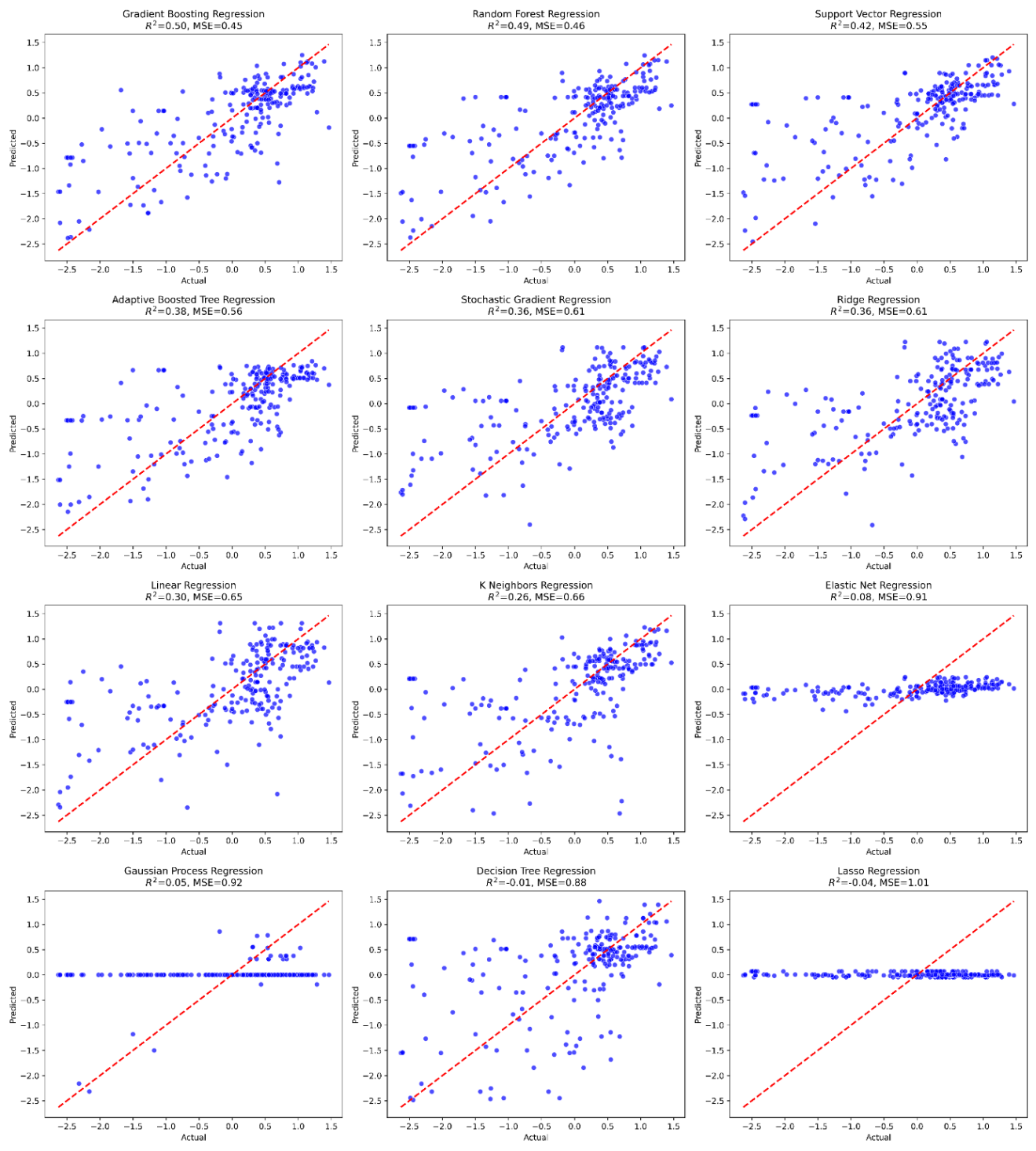


Figure S2. Initial evaluation of regression models from Scikit-Learn, against the full antiviral polymer dataset prior to under sampling, demonstrating poor model performance, particularly at low values for infectivity which are likely to be antiviral (all data presented is scaled using StandardScaler).

Table S1. Summary of regression model evaluation using out of the box models from Scikit-Learn on antiviral polymer data after under-sampling.

| Model name | Avg R2 | Avg MSE | Fold R2 1 | Fold R2 2 | Fold R2 3 | Fold R2 4 | Fold R2 5 |
| --- | --- | --- | --- | --- | --- | --- | --- |
| Support Vector Regressor | 0.653816 | 0.32631 | 0.471005 | 0.699543 | 0.733811 | 0.530164 | 0.834556 |
| Random Forest Regression | 0.608433 | 0.36055 | 0.498391 | 0.681996 | 0.552158 | 0.558704 | 0.750918 |
| Gradient Boosting Regression | 0.560171 | 0.39338 | 0.493301 | 0.703549 | 0.36338 | 0.543355 | 0.697272 |
| K Neighbors Regression | 0.55246 | 0.41989 | 0.356901 | 0.602674 | 0.474735 | 0.605038 | 0.722952 |
| Adaptive Boosted Tree Regression | 0.548303 | 0.41441 | 0.458318 | 0.62474 | 0.446576 | 0.520134 | 0.691746 |
| Ridge Regression | 0.488186 | 0.46423 | 0.354324 | 0.669801 | 0.422202 | 0.334485 | 0.660118 |
| Stochastic Gradient Regression | 0.47487 | 0.48066 | 0.283611 | 0.646031 | 0.378283 | 0.406494 | 0.659932 |
| Linear Regression | 0.434367 | 0.51183 | 0.265322 | 0.614443 | 0.271039 | 0.366918 | 0.654116 |
| Decision Tree Regression | 0.361596 | 0.55196 | 0.684372 | 0.421537 | 0.059563 | 0.117593 | 0.524914 |
| Elastic Net Regression | 0.137592 | 0.8121 | 0.063092 | 0.190993 | 0.007734 | 0.243098 | 0.183043 |
| Gaussian Process Regression | -0.06321 | 0.99089 | -0.0453 | -0.01324 | -0.2695 | 0.001795 | 0.010181 |
| Lasso Regression | -0.10575 | 1.02324 | -0.06537 | -0.021 | -0.39935 | -0.02007 | -0.02293 |


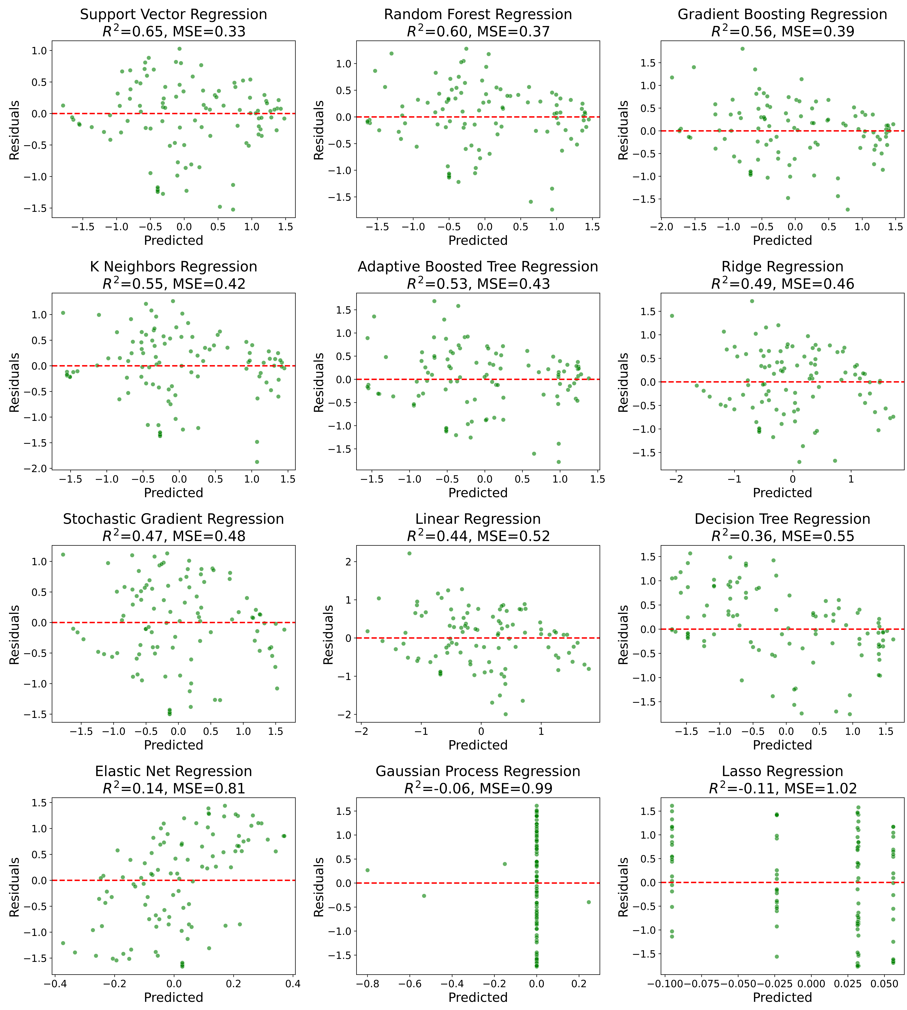


Figure S3. Residuals of predicted values from out of the box regression models from Scikit-Learn on antiviral polymer data after under-sampling.

Table S2. Overview of Mordred descriptors selected by RFECV and used for training regression models on antiviral polymer data.

| Descriptor Label | Description | Additional information |
| --- | --- | --- |
| nBase | basic group count descriptor. | Number of basic nitrogen-containing funcitonal groups |
| AATS3are | averaged ATS descriptor. | Captures how electronegativity (Allred-Rochow) is distributed along atoms 3 bonds apart |
| ATSC8m | centered ATS descriptor. | Captures the distribution of atomic mass along atoms 8 bonds apart |
| ATSC8i | centered ATS descriptor. | Captures the distribution of ionization potential along atoms 8 bonds apart |
| AATSC3c | averaged ATSC descriptor. | Measures correlation of atomic charges (Gasteiger) between atoms 3 bonds apart. |
| AATSC2d | averaged ATSC descriptor. | Measures correlation of sigma electrons between atoms 2 bonds apart. |
| MATS2se | Moran coefficient descriptor. | Captures global correlation of electronegativity (Sanderson scale) between atoms 2 bonds apart. |
| MATS3se | Moran coefficient descriptor. | Captures global correlation of electronegativity (Sanderson scale) between atoms 3 bonds apart. |
| MATS3p | Moran coefficient descriptor. | Captures global correlation of electronegativity (Pauling scale) between atoms 3 bonds apart. |
| GATS4pe | Geary coefficient descriptor. | Captures local differences in electronegativity (Pauling scale) between atoms 4 bonds apart. |
| GATS4are | Geary coefficient descriptor. | Captures local differences in electronegativity (Allred-Rochow scale) between atoms 4 bonds apart. |
| NsssN | AtomTypeEState (‘count’, ‘sssN’) | count of sp³-hybridized nitrogen atoms |
| SsssN | AtomTypeEState (‘sum’, ‘sssN’) | sum of E-State values of all sp³-hybridized nitrogen atoms. |
| MINsCH3 | AtomTypeEState (‘min’, ‘sCH3’) | minimum E-State value of sp³ methyl carbons |
| nHBAcc | hydrogen bond acceptor descriptor(rdkit wrapper). | number of hydrogen bond acceptors |
| IC4 | InformationContent (4) | diversity of atom types and bonding patterns within a 4-bond neighborhood, |
| IC5 | InformationContent (5) | diversity of atom types and bonding patterns within a 5-bond neighborhood, |

Table S3. Overview of default hyperparameters used for training out of the box machine learning regression models from Scikit-Learn.

| Model | Key Hyperparameters |
| --- | --- |
| Linear Regression | copy_X=True, fit_intercept=True, positive=False |
| Ridge Regression | copy_X=True, fit_intercept=True, positive=False, alpha=1.0, random_state=42.0, solver=auto, tol=0.0001 |
| Lasso Regression | copy_X=True, fit_intercept=True, positive=False, alpha=1.0, max_iter=1000.0, random_state=42.0, tol=0.0001, precompute=False, selection=cyclic, warm_start=False |
| Elastic Net Regression | copy_X=True, fit_intercept=True, positive=False, alpha=1.0, max_iter=1000.0, random_state=42.0, tol=0.0001, precompute=False, selection=cyclic, warm_start=False, l1_ratio=0.5 |
| Stochastic Gradient Regression | fit_intercept=True, alpha=0.0001, max_iter=1000.0, tol=0.001, warm_start=False, verbose=0, learning_rate=invscaling, loss=squared_error, n_iter_no_change=5.0, validation_fraction=0.1, average=False, early_stopping=False, epsilon=0.1, eta0=0.01, l1_ratio=0.15, penalty=l2, power_t=0.25, shuffle=True |
| Random Forest Regression | random_state=42.0, warm_start=False, ccp_alpha=0.0, criterion=squared_error, max_features=1.0, min_impurity_decrease=0.0, min_samples_leaf=1.0, min_samples_split=2.0, min_weight_fraction_leaf=0.0, bootstrap=True, n_estimators=100.0, oob_score=False, verbose=0 |
| K Neighbors Regression | algorithm=auto, leaf_size=30.0, metric=minkowski, n_neighbors=5.0, p=2.0, weights=uniform |
| Gradient Boosting Regression | alpha=0.9, random_state=42.0, tol=0.0001, warm_start=False, ccp_alpha=0.0, criterion=friedman_mse, max_depth=3.0, min_impurity_decrease=0.0, min_samples_leaf=1.0, min_samples_split=2.0, min_weight_fraction_leaf=0.0, n_estimators=100.0, verbose=0, learning_rate=0.1, loss=squared_error, subsample=1.0, validation_fraction=0.1 |
| Gaussian Process Regression | alpha=1e-10, random_state=42.0, kernel=1**2 * RBF(length_scale=1), copy_X_train=True, kernel__k1=1**2, kernel__k2=RBF(length_scale=1), kernel__k1__constant_value=1.0, kernel__k1__constant_value_bounds=(0.001, 1000.0), kernel__k2__length_scale=1.0, kernel__k2__length_scale_bounds=(0.01, 100.0), n_restarts_optimizer=10.0, normalize_y=False, optimizer=fmin_l_bfgs_b |
| Support Vector Regression | max_iter=-1.0, tol=0.001, verbose=False, epsilon=0.1, C=1.0, cache_size=200.0, coef0=0.0, degree=3.0, gamma=scale, kernel=rbf, shrinking=True |
| Decision Tree Regression | random_state=42.0, ccp_alpha=0.0, criterion=squared_error, min_impurity_decrease=0.0, min_samples_leaf=1.0, min_samples_split=2.0, min_weight_fraction_leaf=0.0, splitter=best |
| Adaptive Boosted Tree Regression | random_state=42.0, n_estimators=50.0, learning_rate=1.0, loss=linear |

Table S4. Hyperparameters of the three highest performing regression models were tuned using randomized search over the specified distributions, with 5-fold cross-validation and random state=42 for reproducibility.

| Model | Key Hyperparameters |
| --- | --- |
| Random Forest Regression | n_estimators = [int(x) for x in np.linspace(50, 2000, 20)], max_features = ['sqrt', 'log2'], min_samples_split = [2, 3, 5, 10], max_depth = [5, 10, 20, 30] |
| Gradient Boosting Regression | n_estimators = [100, 300, 500, 800, 1000], learning_rate = [0.001, 0.01, 0.05, 0.1, 0.2, 0.3], max_depth = [3, 4, 5, 6, 7, 8, 10], min_samples_split = [2, 5, 10, 15], min_samples_leaf = [1, 2, 4, 6, 8], subsample = [0.6, 0.8, 1.0], max_features = ['sqrt', 'log2', None] |
| Support Vector Regression | C = [0.1, 1, 10, 100], gamma = ['scale', 'auto', 0.01, 0.1, 1], epsilon = [0.01, 0.1, 0.2, 0.5], kernel = ['rbf'] |

Table S5. A list of 119 commercially available methacrylate monomers were clustered into groups of structural similarity using a combination of unsupervised UMAP dimensionality reduction followed by k-means clustering.

| Commercially available methacrylates | CAS | Cluster |
| --- | --- | --- |
| Methacroylcholine Chloride | 5039-78-1 | 0 |
| 3-[[2-(Methacryloyloxy)ethyl]dimethylammonio]propane-1-sulfonate | 3637-26-1 | 0 |
| 3-[[2-(Methacryloyloxy)ethyl]dimethylammonio]propionate | 24249-95-4 | 0 |
| N-[2-(Methacryloyloxy)ethyl]-N,N-dimethylbutan-1-aminium Bis(trifluoromethanesulfonyl)imide | 1616483-28-3 | 0 |
| 2-[[2-(Methacryloyloxy)ethyl]dimethylammonio]acetate | 62723-61-9 | 0 |
| 2-(Methacryloyloxy)-N,N,N-trimethylethanaminium Methyl Sulfate | 6891-44-7 | 0 |
| 4-[[2-(Methacryloyloxy)ethyl]dimethylammonio]butane-1-sulfonate | 6613-65-6 | 0 |
| 2-(Methacryloyloxy)ethyl 2-(Trimethylammonio)ethyl Phosphate | 67881-98-5 | 0 |
| 2-Methacryloyloxyethyl phosphorylcholine | 67881-98-5 | 0 |
| Ethyl Methacrylate | 97-63-2 | 1 |
| (3-Ethyloxetan-3-yl)methyl Methacrylate | 37674-57-0 | 1 |
| Tetrahydrofurfuryl Methacrylate | 2455-24-5 | 1 |
| (2-Oxo-1,3-dioxolan-4-yl)methyl Methacrylate | 13818-44-5 | 1 |
| Allyl Methacrylate | 96-05-9 | 1 |
| Propargyl Methacrylate | 13861-22-8 | 1 |
| Glycidyl Methacrylate | 106-91-2 | 1 |
| 3-Hydroxy-1-methacryloyloxyadamantane | 115372-36-6 | 2 |
| Methacrylic Acid | 79-41-4 | 2 |
| 4-Hydroxyphenyl Methacrylate | 31480-93-0 | 2 |
| Mono-2-(methacryloyloxy)ethyl Phthalate | 27697-00-3 | 2 |
| 2-(tert-Butylamino)ethyl Methacrylate | 3775-90-4 | 2 |
| 2-Aminoethyl Methacrylate | 2420-94-2 | 2 |
| 2,2,6,6-Tetramethyl-4-piperidyl Methacrylate | 31582-45-3 | 2 |
| 2-[2-Hydroxy-5-[2-(methacryloyloxy)ethyl]phenyl]-2H-benzotriazole | 96478-09-0 | 2 |
| 3-Chloro-2-hydroxypropyl Methacrylate | 13159-52-9 | 2 |
| 2-Hydroxybutyl Methacrylate | 13159-51-8 | 2 |
| i-Hydroxypropyl Methacrylate | 27813-02-1 | 2 |
| 6-Hydroxyhexyl Methacrylate | 13092-57-4 | 2 |
| 2-Hydroxyethyl Methacrylate | 868-77-9 | 2 |
| Polyethylene Glycol Monomethyl Ether Methacrylate (n=approx. 9) | 26915-72-0 | 2 |
| Polyethylene Glycol Monomethacrylate (n=approx. 5) | 25736-86-1 | 2 |
| Polyethylene Glycol Monomethyl Ether Methacrylate (n=approx. 13) | 26915-72-0 | 2 |
| Polyethylene Glycol Monomethyl Ether Methacrylate (n=approx. 23) | 26915-72-0 | 2 |
| Polyethylene Glycol Monomethyl Ether Methacrylate (Mw.=ca. 2100) | 26915-72-0 | 2 |
| n-Hydroxypropyl Methacrylate | 27813-02-1 | 2 |
| Isobornyl Methacrylate | 7534-94-3 | 3 |
| 2-[(3r,5r,7r)-Adamantan-1-yl]propan-2-yl Methacrylate | 279218-76-7 | 3 |
| 2-Cyclohexylpropan-2-yl Methacrylate | 186585-56-8 | 3 |
| Cyclohexyl Methacrylate | 101-43-9 | 3 |
| 1-Adamantyl Methacrylate | 16887-36-8 | 3 |
| 2-Ethyl-2-methacryloyloxyadamantane | 209982-56-9 | 3 |
| 2-Isopropyl-2-methacryloyloxyadamantane | 297156-50-4 | 3 |
| 2-Methacryloyloxy-2-methyladamantane | 177080-67-0 | 3 |
| Dicyclopentanyl Methacrylate | 34759-34-7 | 3 |
| 1-Isopropylcyclopentyl Methacrylate | 1149760-04-2 | 3 |
| 1-Ethylcyclopentyl Methacrylate | 266308-58-1 | 3 |
| 1-Ethylcyclohexyl Methacrylate | 274248-09-8 | 3 |
| 1-Methylcyclopentyl Methacrylate | 178889-45-7 | 3 |
| Mevalonic Lactone Methacrylate | 177080-66-9 | 3 |
| 2-(Dimethylamino)ethyl Methacrylate | 2867-47-2 | 4 |
| 2-(Diethylamino)ethyl Methacrylate | 105-16-8 | 4 |
| 3-(Dimethylamino)propyl Methacrylate | 20602-77-1 | 4 |
| 2-(Diisopropylamino)ethyl Methacrylate | 16715-83-6 | 4 |
| 2-(2-Pyridinyldithio)ethyl Methacrylate | 910128-59-5 | 4 |
| 1,2,2,6,6-Pentamethyl-4-piperidyl Methacrylate | 68548-08-3 | 4 |
| 2-Morpholinoethyl Methacrylate | 2997-88-8 | 4 |
| 2-(2-Oxopyrrolidin-1-yl)ethyl Methacrylate | 946-25-8 | 4 |
| 2-[[(4-Nitrophenoxy)carbonyl]oxy]ethyl Methacrylate | 599179-75-6 | 4 |
| N-Succinimidyl Methacrylate | 38862-25-8 | 4 |
| 2-Isocyanatoethyl Methacrylate | 30674-80-7 | 4 |
| 2-(Diisopropylamino)ethyl methacrylate | 16715-83-6 | 4 |
| 2,2,3,4,4,4-Hexafluorobutyl Methacrylate | 36405-47-7 | 5 |
| 2,2,3,3,4,4,4-Heptafluorobutyl Methacrylate | 13695-31-3 | 5 |
| 2,2,2-Trifluoroethyl Methacrylate | 352-87-4 | 5 |
| 1H,1H,2H,2H-Nonafluorohexyl Methacrylate | 1799-84-4 | 5 |
| 1H,1H,2H,2H-Nonafluorohexyl Methacrylate | 1799-84-4 | 5 |
| 1H,1H,5H-Octafluoropentyl Methacrylate | 355-93-1 | 5 |
| 2,2,3,3,3-Pentafluoropropyl Methacrylate | 45115-53-5 | 5 |
| 1H,1H,2H,2H-Tridecafluoro-n-octyl Methacrylate | 2144-53-8 | 5 |
| 2,2,3,3-Tetrafluoropropyl Methacrylate | 45102-52-1 | 5 |
| 2,2,3,3,4,4,5,5,6,6,7,7-Dodecafluoroheptyl Methacrylate | 2261-99-6 | 5 |
| Sodium Methacrylate | 5536-61-8 | 6 |
| 3-Sulfopropyl Methacrylate | 31098-21-2 | 6 |
| 2-Ethoxyethyl Methacrylate | 2370-63-0 | 6 |
| Diethylene Glycol Monomethyl Ether Methacrylate | 45103-58-0 | 6 |
| 2-Methoxyethyl Methacrylate | 6976-93-8 | 6 |
| Ethylene Glycol Monoacetoacetate Monomethacrylate | 21282-97-3 | 6 |
| Polyethylene Glycol Monomethyl Ether Methacrylate (n=approx. 4) | 26915-72-0 | 6 |
| 2-[(2-Bromo-2-methylpropanoyl)oxy]ethyl Methacrylate | 213453-08-8 | 6 |
| Triethylene glycol methyl ether methacrylate | 24493-59-2 | 6 |
| Benzyl Methacrylate | 2495-37-6 | 7 |
| 2-Phenylethyl Methacrylate | 3683-12-3 | 7 |
| 9-Anthrylmethyl Methacrylate | 31645-35-9 | 7 |
| 4-Benzoylphenyl Methacrylate | 56467-43-7 | 7 |
| Phenyl Methacrylate | 2177-70-0 | 7 |
| Pentafluorobenzyl Methacrylate | 114859-23-3 | 7 |
| Pentafluorophenyl Methacrylate | 13642-97-2 | 7 |
| 2-Phenoxyethyl Methacrylate | 10595-06-9 | 7 |
| Furfuryl Methacrylate | 3454-28-2 | 7 |
| Butyl Methacrylate | 97-88-1 | 8 |
| 2-Ethylhexyl Methacrylate | 688-84-6 | 8 |
| Hexyl Methacrylate | 142-09-6 | 8 |
| Dodecyl Methacrylate | 142-90-5 | 8 |
| 11-[4-(4-Butylphenylazo)phenoxy]undecyl Methacrylate | 942230-11-7 | 8 |
| Stearyl Methacrylate | 32360-05-7 | 8 |
| 2-Methacryloyloxyethyl Thioctate | 126449-41-0 | 8 |
| 3-(Ethoxydimethylsilyl)propyl Methacrylate | 13731-98-1 | 8 |
| (Triethoxysilyl)methyl Methacrylate | 5577-72-0 | 8 |
| 2-(Trimethylsilyloxy)ethyl Methacrylate | 17407-09-9 | 8 |
| 3-(Trimethoxysilyl)propyl Methacrylate | 2530-85-0 | 8 |
| 3-[Tris(trimethylsilyloxy)silyl]propyl Methacrylate | 7096-07-0 | 8 |
| 3-(Triethoxysilyl)propyl Methacrylate | 21142-29-0 | 8 |
| 3-[Diethoxy(methyl)silyl]propyl Methacrylate | 65100-04-1 | 8 |
| 3-[Dimethoxy(methyl)silyl]propyl Methacrylate | 14513-34-9 | 8 |
| 3-(Chlorodimethylsilyl)propyl Methacrylate | 24636-31-5 | 8 |
| [Dimethoxy(methyl)silyl]methyl Methacrylate | 121177-93-3 | 8 |
| Methyl Methacrylate | 80-62-6 | 9 |
| Isobutyl Methacrylate | 97-86-9 | 9 |
| Isopropyl Methacrylate | 4655-34-9 | 9 |
| tert-Butyl Methacrylate Monomer | 585-07-9 | 9 |
| sec-Butyl Methacrylate | 2998-18-7 | 9 |
| 1,1,1,3,3,3-Hexafluoroisopropyl Methacrylate | 3063-94-3 | 9 |
| 1-Ethoxyethyl Methacrylate | 51920-52-6 | 9 |
| 2-Oxotetrahydrofuran-3-yl Methacrylate | 195000-66-9 | 9 |
| 2-Oxo-2-[(5-oxo-4-oxatricyclo[4.2.1.03,7]nonan-2-yl)oxy]ethyl Methacrylate | 347886-81-1 | 9 |
| 2-Oxohexahydro-2H-3,5-methanocyclopenta[b]furan-6-yl Methacrylate | 254900-07-7 | 9 |
| 5-Oxotetrahydrofuran-3-yl Methacrylate | 130224-95-2 | 9 |
| Trimethylsilyl Methacrylate | 13688-56-7 | 9 |
| Methacrylic Anhydride | 760-93-0 | 9 |

Table S6. Overview of molecular descriptors used with Morgan fingerprints for unsupervised clustering of methacrylates via UMAP dimensionality reduction and k-means clustering.

| Descriptor label | Description |
| --- | --- |
| SLogP | Wildman-Crippen LogP descriptor |
| TopoPSA | topological polar surface area |
| nN | number of N atoms |
| nO | number of O atoms |
| nRot | rotatable bonds count |
| nHBDon | number of hydrogen bond donors |
| nHBAcc | number of hydrogen bond acceptors |

Table S7. A summary of outlier polymers from model evaluation for which the RF model has the lowest predictive accuracy. Including polymer composition (mol %), actual infectivity, predicted infectivity, and the absolute error of the predicted values.

| Polymer Composition (mol %) | Actual Infectivity | Predicted Infectivity | Absolute Error |
| --- | --- | --- | --- |
| Triethylene glycol methyl ether methacrylate(40%)-Benzyl Methacrylate(34%)-2-(Diisopropylamino)ethyl Methacrylate(27%) | 11.5 | 48.8 | 37.3 |
| Polyethylene Glycol Monomethyl Ether Methacrylate (n=. 4)(27%)-3-Sulfopropyl Methacrylate(54%)-2-Methacryloyloxyethyl phosphorylcholine(19%) | 13.4 | 49.2 | 35.8 |
| Polyethylene Glycol Monomethyl Ether Methacrylate (n=4)(28%)-2-(Diethylamino)ethyl Methacrylate(51%)-2-Methacryloyloxyethyl phosphorylcholine(21%) | 53.6 | 23.2 | 30.4 |
| Triethylene glycol methyl ether methacrylate(67%)-2-(Diethylamino)ethyl Methacrylate(24%)-3-Sulfopropyl Methacrylate(9%) | 103.6 | 75.6 | 28.0 |
| 2-(Diisopropylamino)ethyl Methacrylate(34%)-3-Sulfopropyl Methacrylate(49%)-2-Methacryloyloxyethyl phosphorylcholine(17%) | 71.2 | 43.8 | 27.5 |
| Polyethylene Glycol Monomethyl Ether Methacrylate (n=approx. 4)(7%)-Benzyl Methacrylate(14%)-2-(Diethylamino)ethyl Methacrylate(34%)-3-[[2-(Methacryloyloxy)ethyl]dimethylammonio]propane-1-sulfonate(45%) | 49.9 | 25.78 | 24.2 |
| Polyethylene Glycol Monomethyl Ether Methacrylate (n=4)(56%)-2-(Diethylamino)ethyl Methacrylate(35%)-2-Methacryloyloxyethyl phosphorylcholine(9%) | 93.9 | 71.7 | 22.2 |
| Polyethylene Glycol Monomethyl Ether Methacrylate (n=4)(19%)-Dodecyl Methacrylate(9%)-2-(Diethylamino)ethyl Methacrylate(50%)-2-Methacryloyloxyethyl phosphorylcholine(21%) | 5.2 | 18.0 | 12.8 |
| Polyethylene Glycol Monomethyl Ether Methacrylate (n=approx. 4)(8%)2-(Diethylamino)ethyl Methacrylate(27%)-3-Sulfopropyl Methacrylate(20%)-3-[[2-(Methacryloyloxy)ethyl]dimethylammonio]propane-1-sulfonate(45%) | 33.3 | 45.6 | 12.3 |
| Triethylene glycol methyl ether methacrylate(39%)-2-(Diethylamino)ethyl Methacrylate(24%)-3-Sulfopropyl Methacrylate(37%) | 78.7 | 66.8 | 11.9 |


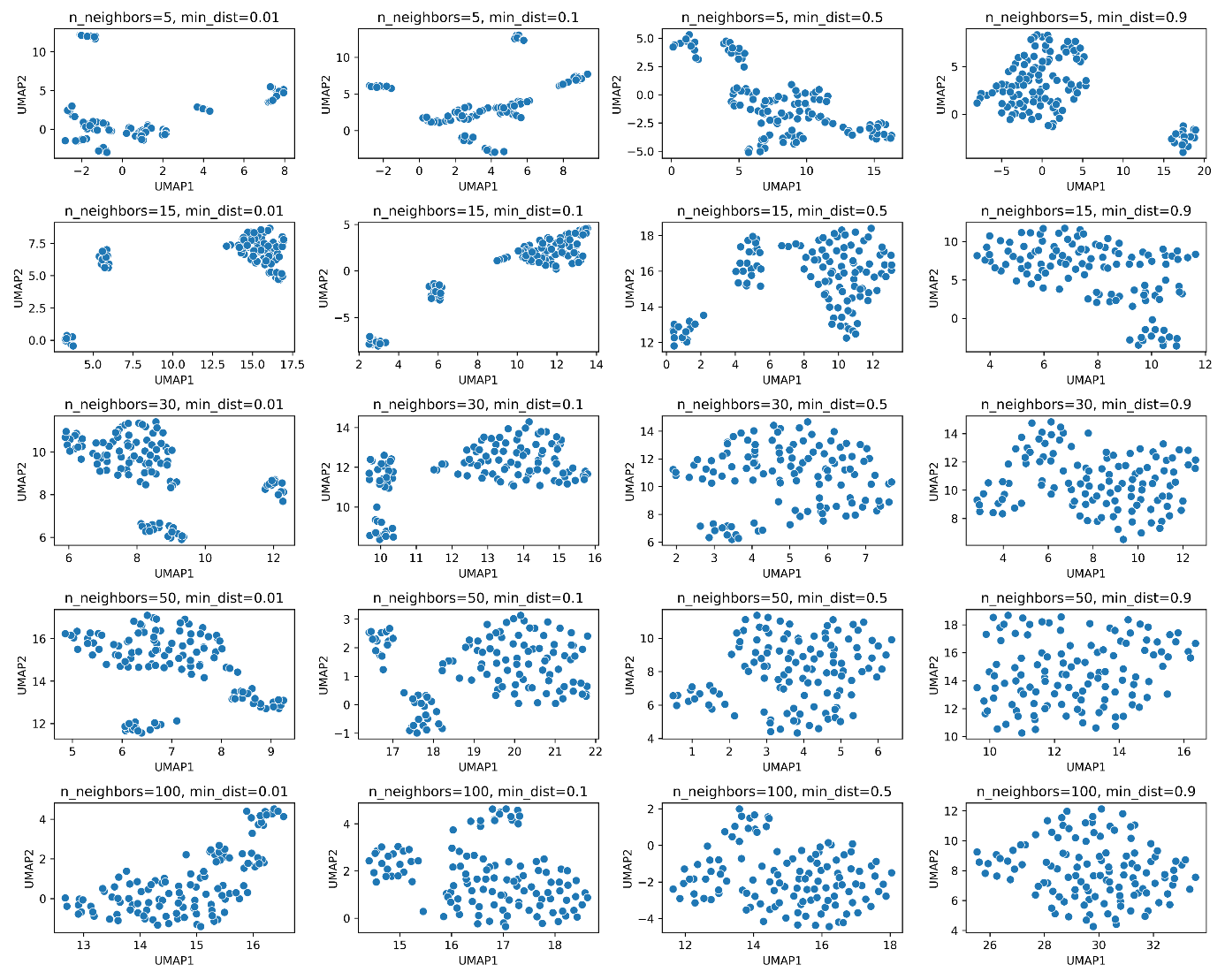


Figure S4. Optimization of hyperparameters for UMAP for dimensionality reduction of methacrylate descriptors and fingerprints.


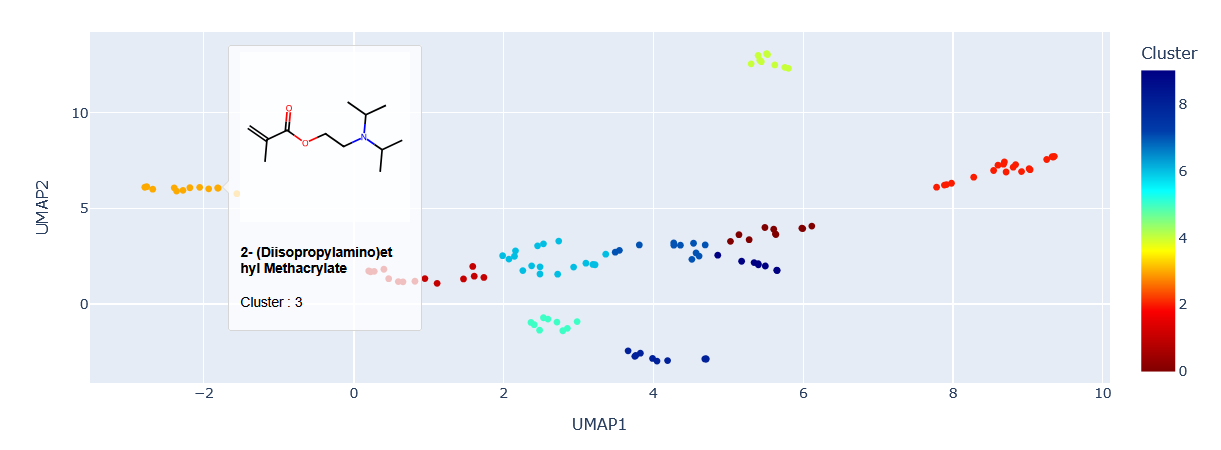


Figure S5. Molplotly was used to make a dynamic plot to easily assess monomer clustering during unsupervised lear
